# The State of Web Accessibility for People with Cognitive Disabilities: A Rapid Evidence Assessment

**DOI:** 10.3390/bs12020026

**Published:** 2022-01-26

**Authors:** Sara Gartland, Paul Flynn, Maria Ana Carneiro, Greg Holloway, Jose de Sousa Fialho, Joe Cullen, Emma Hamilton, Amy Harris, Clare Cullen

**Affiliations:** 1 School of Education, National University of Ireland Galway, H91 TK33 Galway, Ireland; paul.flynn@nuigalway.ie; 2 CEPCEP, Universidade Católica Portuguesa, 1649 023 Lisbon, Portugal; mianacarneiro2@gmail.com (M.A.C.); jasousafialho@gmail.com (J.d.S.F.); 3 ARCOLA Research LLP, London E8 2NW, UK; gholloway2@arcola-research.co.uk (G.H.); jcullen@arcola-research.co.uk (J.C.); ehamilton@arcola-research.co.uk (E.H.); aharris@arcola-research.co.uk (A.H.); ccullen@arcola-research.co.uk (C.C.)

**Keywords:** web accessibility, cognitive disability, cognitive impairment

## Abstract

Increased digitisation of day-to-day activities was occurring prior to the COVID-19 pandemic. The pandemic only accelerated the virtual shift, making web accessibility an urgent issue, especially for marginalised populations. Despite decades of work to develop, refine, and implement web accessibility standards, people with cognitive disabilities regularly experience many barriers to web accessibility. To inform ongoing work to improve web accessibility for people with cognitive disabilities, a systematic review was conducted. The main question guiding this review is: what are the state-of-the-art of interventions that support web accessibility for citizens, 9 years of age and up, living with cognitive impairment? A set of 50 search strings were entered into three academic databases: SCOPUS, ProQuest, and Web of Science. Systematic screening procedures narrowed the search returns to a total of 45 included papers. A data analysis revealed themes associated with the lived experiences of people with cognitive disabilities, tools for improving web accessibility, and methodological best practices for involving people with cognitive disabilities in research. These findings have immediate implications for ongoing research and the development of meaningful solutions to the problem of web accessibility for people with cognitive disabilities.

## 1. Introduction

The COVID-19 pandemic sharply increased the embeddedness of digital technologies in daily life which simultaneously amplified existing social, educational, and economic inequities. Prior to the pandemic, research highlighted the importance of better understanding the barriers to technology use experienced by people with cognitive disabilities [1,2,3], a group that is often marginalised in society and in research [4]. Thus, the COVID-19 pandemic and subsequent expansion of digital technologies associated with education, wellness, and employment makes the need to understand how people with cognitive disabilities interact with such technologies increasingly urgent.

A crucial first step is to develop an understanding of the current state of web accessibility for people with cognitive disabilities. Thus, the purpose of this review of the state of web accessibility for people with cognitive disabilities is to conduct baseline research to understand the factors and processes that inhibit access to online content and services for people with cognitive impairments. To aid in framing this review, we now provide definitions of the key constructs that informed this work.

### 1.1. People with Cognitive Disabilities

The labels cognitive disability and cognitive impairment are used to describe a wide range of issues associated with mental processes. Such terms are widely used, and definitions can vary greatly. Different populations also favour some descriptors over others. To provide focus to this review and assist in interpretation of the results, we provide a concise definition and use a singular term. People with cognitive disabilities (PwCDs) experience difficulties with memory, executive function, reasoning, attention, language, literacy, perception, emotional expression, and comprehension for a variety of reasons including, but not limited to, intellectual disabilities, brain injuries, dementia, and other age-related issues [5,6].

### 1.2. Web Accessibility

Web accessibility and web usability are related terms with overlapping components. Often web accessibility studies involve some usability testing. However, usability is not a full picture of accessibility. For the purpose of this review, we focus on web accessibility, which “addresses discriminatory aspects related to equivalent user experience on the web” [7] (p. 3) for all people. Web accessibility can include considerations of usability, which “is about effective, efficient, and satisfying design of websites and mobile applications” (p. 4). Additionally, web accessibility is inclusive of websites, web apps, web-based tools and platforms, and other internet connected technologies.

### 1.3. Web Accessibility for People with Cognitive Disabilities

The World Wide Web Consortium (W3C), an international community committed to developing open standards for web development, launched the Web Accessibility Initiative (WAI) to improve web accessibility for people with disabilities. Through WAI, W3C developed the Web Content Accessibility Guidelines (WCAG). W3C’s Cognitive and Learning Disability (COGA) Task Force identified which of the current guidelines support users with cognitive disabilities [8]. Additionally, the COGA Task Force has published recommended scenarios for investigation in user group research around eight specific cognitive disabilities: Dyslexia, Aphasia, Non-Verbal, Down Syndrome, Autism, Dyscalculia, Aging-Related Cognitive Decline, and Attention Deficit Disorder (ADD/ADHD). These recommendations shape our definition of web accessibility for PwCDs. For us, web accessibility for PwCDs means that web users with cognitive disabilities can navigate and engage with websites, web tools, and web technologies to accomplish tasks associated with health, education, and employment, to communicate, to use the web of things and web systems, and to access critical information.

## 2. Purpose and Review Questions

Against the background of the accelerated digitisation of almost all aspects of life, the LIVE-IT project, a multi-jurisdictional (Greece, Ireland, Portugal, and the UK) research consortium with a focus on web accessibility for people with cognitive disabilities has begun work to further explore web accessibility issues. In order to address the urgent needs associated with web accessibility for people with cognitive disabilities, research partners will develop co-design labs to lead improvements and innovations in web tools and platforms. The overarching goal for the LIVE-IT project is to aggregate and valorise the insights and findings of different ‘research lenses’ on the relationship between cognitive disabilities and digital inclusion into actionable experimentation spaces. Additionally, the LIVE-IT project aims to support inclusive accessibility by tailoring design to the settings in which people with cognitive disabilities engage with digital technologies in their everyday lives, or their ‘lifeworld’ [9]. The review reported here was conducted to support the development of the co-design labs. The review was guided by one overarching question and several sub-questions. The overarching review question is:

- What are the state-of-the-art of interventions that support web accessibility for citizens, 9 years of age and up, living with cognitive impairment?

Furthermore, six sub-questions add focus to the overarching question:

- What are examples of good practices, ‘interventions,’ including examples of platforms and tools, that have the potential to support inclusive web accessibility for people with cognitive disabilities?
- What is the impact of successful initiatives, interventions, or programmes on supporting inclusive web accessibility for people with cognitive impairment?
- What are the similarities and differences between successful initiatives, interventions, or programmes supporting web accessibility for people with cognitive impairment in the different sectors (i.e., education, health, commerce, employment, etc.)?
- What are the drivers and barriers, or supports and constraints, for web accessibility for people with cognitive impairment?
- What are the key characteristics of successful initiatives, interventions, or programmes on supporting inclusive web accessibility for the population under study?
- What recommendations can be made for the future initiatives that wish to support web accessibility for citizens living with cognitive impairment?

Prior to reporting the results of the rapid evidence assessment, we now provide a brief overview of policies and guidelines that shape the nature of web accessibility, both in general and for people with cognitive disabilities.

## 3. Background

### 3.1. Relevant Policies and Guidelines

One policy and one set of guidelines appear to serve as the framework for the state policies reviewed. The United Nations (UN) Convention on Rights of Persons with Disabilities (CRPD) is the overarching policy that both informs and drives the EU and individual state policies [10,11,12,13,14,15,16,17]. Additionally, the World Wide Web Consortium’s (W3C’s) Web Content Accessibility Guidelines (WCAG) serve as a foundation for EU and individual state policies. An initial review of policy and guideline articles revealed that only parts of the CRPD and the WCAG are specifically included in the EU and individual state policies. Namely, the CRPD and the WCAG are more comprehensive and rigorous in their targets for accessibility than the EU and individual state policies.

**Figure 1:**
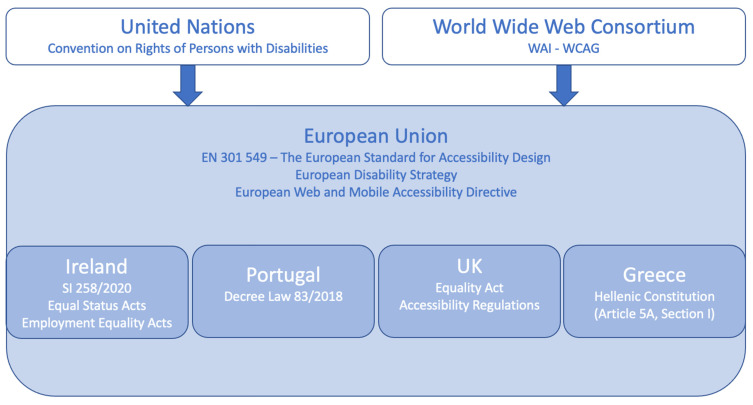
Major web accessibility policies and guidelines.

Figure 1, on the following page, displays how the policies and guidelines are related. Recall that the LIVE-IT Project, the H2020 pilot project for which this review is being conducted, involves four specific jurisdictions: Ireland, Portugal, the UK, and Greece. Therefore, the policies framing this review and the work within the LIVE-IT Project are the policies within the four jurisdictions listed.

**Figure 2:**
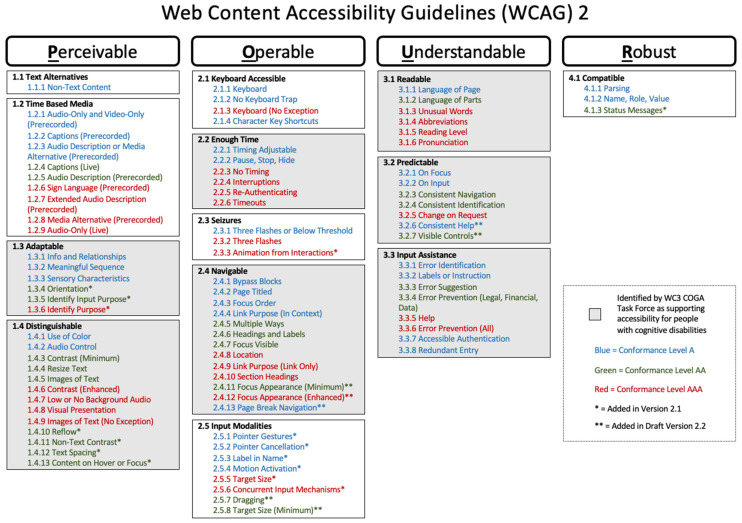
WCAG 2.0 [8,18].

The dominant set of guidelines for web accessibility, broadly speaking, are the W3C’s WCAG [18]. These guidelines have been revised since their introduction. The WCAG 1.0 were released in 1999 and subsequently incorporated into policy recommendations around the world. A decade later, the WCAG 2.0, which included additions and revisions to the WCAG 1.9, were released. The WCAG 2.0 are what appear in most policies, and are organised around the acronym POUR [18], as illustrated in Figure 2, on the following page. POUR stands for Perceivable-Operable-Understandable-Robust, and these descriptors link to specifications for web pages and web apps that support accessibility at three different levels. The WCAG levels are labelled as A, AA, and AAA. Most policies require WCAG compliance at the AA level, which means that most policies do not require the highest level of accessibility. Figure 2 also captures the additions to the WCAG in versions 2.1 and 2.2. It is important to note that the WCAG is an open-source-like set of guidelines, meaning that all discussion around changes to the guidelines are public.

One major critique of the WCAG has been the lack of accessibility specifications to support people with cognitive disabilities [19,20]. W3C as an organisation recognised this problem and developed the COGA Task Force, whose mission is to improve current accessibility standards and recommend new accessibility standards focused on people with cognitive disabilities. Figure 2 also highlights the specifications in the WCAG 2.0 that support accessibility for people with cognitive disabilities as explained by the W3C COGA Task Force [8,18]. However, a limited volume of published research exists on how effective the existing standards are for people with cognitive disabilities.

### 3.2. Prior Reviews

While developing the methods for this review, two related reviews were located; one focused on web usability for people with cognitive disabilities [21]. We aim to expand on this knowledge by addressing accessibility, rather than just usability. As noted above, web usability is not synonymous with web accessibility. A second, focused on web accessibility, broadly speaking, not specifically for people with cognitive disabilities, with respect to educational websites [22]. Our review aims to specifically address PwCDs.

Additionally, in the peer-review process, four related literature reviews were located [23,24,25,26]. Borg, et al. [23] focused on the accessibility of electronic communication for PwCDs. The review presented in this paper expands on their findings that accessibility needs for PwCDs are diverse by expanding the search to include more than just electronic communication. Petrie, et al. [24] critically examined literature on technologies designed for people with disabilities and the elderly. Our review differs in that it includes children and younger adults in our review, and it focuses on web-based technologies. Mack, et al. [25] reviewed CHI and ASSETS papers focused on accessibility, without limiting the search to accessibility to PwCDs. They highlighted trends in overrepresentation and underrepresentation, which support our decision to focus specifically on PwCDs in this review. Finally, Dell, et al. [26] conducted a review of literature on accessibility in Rich Internet Applications. Again, the review presented in this paper will build on such work by investigating accessibility for PwCDs.

## 4. Review Methods

This systematic review of peer reviewed literature was carried out using rapid evidence assessment methods [27]. The protocol for this review was submitted to PROSPERO, the International Prospective Register of Systematic Reviews. Following the search, results were subjected to screening and quality appraisal. The processes for searching, screening, including, or excluding papers, and quality appraisal, as set out by the protocol, are detailed below.

### 4.1. Search and Initial Screening Procedures

A total of 50 search strings, see Appendix B, were developed for use in databases. The search strings were created by reviewing relevant policy and guideline documents mentioned above, conducting preliminary searches in Google Scholar and Google, and consulting the methodology sections of related reviews referred to previously. After developing the search strings, the strings were entered into the following databases: Web of Science, SCOPUS, and ProQuest. Within the Web of Science, SCOPUS, and ProQuest databases, fifteen-year time limited versions of the 50 search strings were entered. Additionally, searches were restricted to peer reviewed results, and each search was set to “all fields.” Internet searching through Google Scholar was carried out as well as forward and backward tracking of citations from studies returned in the database search results.

Following the PRISMA_P standard reporting process for systematic reviews, all results were imported to Mendeley, and duplicates were removed. Initially, we proposed that we include ERIC and EBSCOhost, but upon closer inspection, it was discovered that the ERIC and EBSCOhost searches covered the same databases as the ProQuest searches. Thus, the search returns from ERIC and EBSCOhost were immediately labelled as duplicates and not imported to Mendeley. Additionally, these search returns are not counted in any totals reported. After the removal of duplicates, titles and abstracts were reviewed by-hand to screen for relevance. For example, some of the search strings returned papers reporting on medical trials. Such papers were removed immediately. This is considered a separate step from applying the inclusion and exclusion criteria. For example, articles reporting on drug trials were not relevant and excluded in this first round of screening.

### 4.2. Inclusion and Exclusion Criteria

After removal of duplicates and the initial screening for relevance, the remaining titles and abstracts were screened a second time using the inclusion and exclusion criteria (see Table 1, below). The inclusion and exclusion criteria were developed using the Population, Intervention, Comparison, Outcome (PICO) model as an initial framework. We say initial, because the research team found it necessary to add categories to the inclusion and exclusion criteria. The added categories capture the multi-jurisdictional nature of the broader project funding of this review and the emergent nature of the work in this field: publication, geographic location, and study design.

#### 4.2.1. Publication

Searches were limited to a 15-year window: 2006–2021. The research team decided on the 15-year window due to the introduction of the smartphone occurring in 2007. The first iPhone was released in 2007, which instigated a rapid increase in both hard-line and mobile web accessibility for the general population. Thus, we expected that a potentially sharp increase in accessibility related literature might have occurred around, or just after, 2007. Since we could not locate literature reviews specifically addressing web accessibility for people with cognitive disabilities, we felt it important to capture any related literature from at least 2007. Using a 15-year window allowed us to capture the time leading up to and immediately following this watershed date.

#### 4.2.2. Geographic Location

All geographic locations were considered for this review, meaning that no studies were excluded based on geographic location. However, due to the fact that this review informs the LIVE-IT Project activities, which take place in four specific locations, the authors made efforts to locate studies from the geographic locations in which LIVE-IT activities would be carried out. Thus, when conducting supplemental searches and/or tracking references, preference was granted to the work carried out in the European Union and the four countries represented by the research consortium: Greece, Ireland, Portugal, and the United Kingdom.

#### 4.2.3. Study Design

In terms of study design, we included or excluded papers based on the study population. First, the study had to involve people with cognitive disabilities. Second, if the study was entirely automated, meaning there were no human participants, the study was excluded.

#### 4.2.4. Population

The review focused on citizens 9 years of age and up who live with cognitive impairment. The lower age limit of 9 years of age was set to capture the year, typically, prior to the transition from primary school to secondary school across the multiple jurisdictions represented in this research partnership. Often, children are diagnosed with cognitive disabilities in secondary school due to the prevalence of high stakes testing during that time. No upper age limit was set, because the need for web accessibility when living with cognitive impairment does not diminish or stop at any particular age. Studies that did not provide evidence of sufficient data focused exclusively on citizens with cognitive impairments were excluded from this review. For example, if a study listed participants with disabilities, but did not disaggregate data enough to determine which results applied to people with cognitive disabilities versus physical disabilities, the study was excluded.

#### 4.2.5. Intervention

Again, the only criteria for interventions focused on the study population. First, the intervention had to involve people with cognitive disabilities. Second, if results were not reported in a way that made it clear what data were collected from people with cognitive disabilities, the paper, or the study, was excluded.

#### 4.2.6. Comparison

Because all study designs were considered for this review, we did not require a comparator or control group. We expected studies in this area to be qualitative or quasi-experimental in nature. Excluding such studies when no prior reviews of research on web accessibility for people with cognitive disabilities could be located would be too restrictive.

#### 4.2.7. Outcome

Data and results were reported in the article for inclusion in this review. The data and the results did not need to be of any particular form. Quantitative, qualitative, and mixed-methods data were all included in this review.

### 4.3. Application of Inclusion and Exclusion Criteria

**Figure 3:**
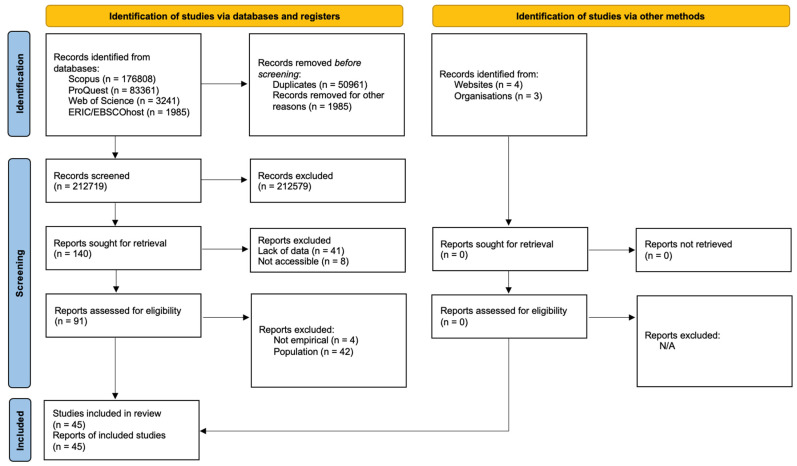
PRISMA-P flow chart.

As illustrated in the PRISMA-P diagram (see Figure 3, below), the screening process began after removal of duplicates. Screening of titles and abstracts using the inclusion and exclusion criteria reduced the total number of records to 140. At this point, the decision was made to access the PDFs of the 140 articles to further assess the studies. While accessing the PDFs, some articles were excluded prior to reading the full paper. For example, if the PDF included a paper within conference proceedings that described a study but not the results. In some cases, the PDFs were not accessible.

Thus, 91 articles were screened in full for potential inclusion. The majority of the articles excluded at this stage were due to the study population. In most cases, the study population was ultimately not described in enough detail to determine which results, if any, specifically related to people with cognitive disabilities. After screening, a total of 45 studies were accepted for inclusion in the review.

### 4.4. Quality Appraisal

This review relied on the Critical Appraisal Skills Programme (CASP) checklists [28] for quality appraisal of the 45 studies included in the review. After applying the inclusion and exclusion criteria above, and before extracting data for the review, the studies were evaluated using the CASP checklist. Appendix A displays the full quality appraisal for all 45 studies included in the review. No studies were excluded based on the CASP quality appraisal process.

### 4.5. Data Extraction and Analysis

Because the majority of the studies reviewed were qualitative or mixed methods, the data extraction and analysis process relied on thematic analysis [29]. All 45 papers selected for inclusion in the review were read in full three times: once during the screening process to ensure appropriateness for inclusion, once to record study design and findings, and once to review findings and contributions in light of other studies included in the review. Data extracted aligned with the PICO structure of the inclusion and exclusion criteria and was stored in an Excel spreadsheet. All data were then reviewed to qualitatively establish themes and connections across the data extracted from the studies. Themes and connections emerged around findings and methodology, which will now be presented in more detail.

## 5. Results

The overarching question guiding this review is: what are the state-of-the-art of interventions that support web accessibility for citizens, 9 years of age and up, living with cognitive impairment? We begin answering this question by highlighting some descriptive trends related to study design and focus prior to delving into the specifics of the themes that emerged with respect to study findings. Finally, we explore three key themes emerging from our analysis in three separate sub-sections, which follow this introduction.

As mentioned in the previous section, 45 papers were included in this review. We noticed some descriptive trends with respect to study design as we made decisions about which CASP checklist to use. This is important, because there are different CASP checklists for qualitative and experimental studies, and the checklist for experimental studies focuses on the decisions related to control group design. The studies included were primarily qualitative or quasi-experimental (N = 31). In the few instances where an experimental design (N = 14) was used, the sample sizes tended to be small, or evidence of randomisation was limited. Thus, the CASP checklist for qualitative research was used for all studies, and as noted in the previous section, no studies were excluded based on the CASP checklist results. Table 2 and Table 3 below provide an overview of the results by study design: qualitative, mixed methods, and quasi-experimental (Table 2) and experimental (Table 3).

Note that in Table 2 and Table 3, which are a reorganisation of the full CASP table provided in Appendix A, some studies received less-than-stellar marks. For example, three studies in total were rated as having an insufficiently rigorous data analysis. However, they were included in this review. This is because we want to provide an accurate state-of-the-art, and there is something to be learned from studies that may be starting to do the work of improving web accessibility for PwCDs but may miss the mark in terms of design and reporting.

**Figure 4:**
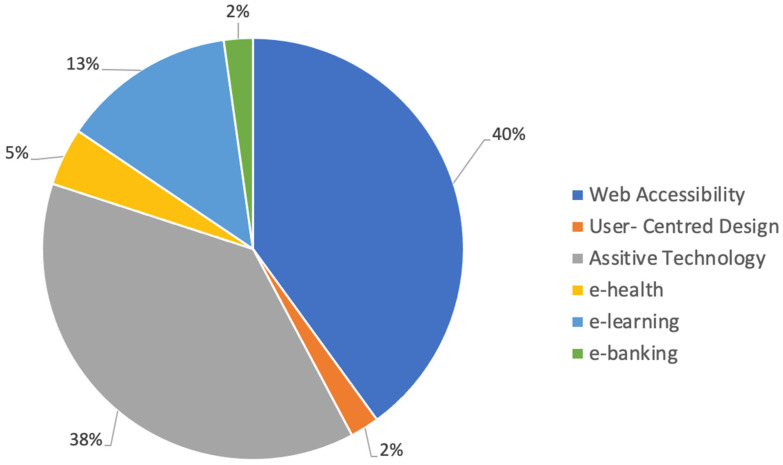
Overview of studies included by search string topic.

Figure 4, on the next page, illustrates a second set of descriptive findings for the full list of 45 studies included in this review: an overview of the studies by search string group. Appendix B includes the full list of search strings used for this systematic review. Within Mendeley, all returns were grouped by search string and database, which made it easy to determine which search strings produced each of the 45 studies included. Recall that 50 search strings were used. Therefore, with 45 studies, we had fewer studies than search strings, which prompted the grouping of search strings by topic. Six topics emerged from this exercise: web accessibility, user-centred design, assistive technology, e-health, e-learning, and e-banking. As shown in Figure 4, 40% of the studies reviewed addressed web accessibility for PwCDs, broadly defined, while 60% of the studies reviewed addressed more specific areas of web accessibility for PwCDs.

Through our analysis, we learned that the state-of-the-art of interventions that support web accessibility for citizens, 9 years of age and up, living with cognitive impairment is still fraught with barriers. Our findings suggest that an increased and more authentic inclusion of PwCDs in the research is needed to continue improving the state-of-the art. To support this claim, we present the results of this systematic review via three overarching themes. First, the studies reviewed provide a clear view of the barriers to web accessibility experienced by people with cognitive disabilities. Second, some studies made contributions in the form of producing or assessing tools for web accessibility for people with cognitive disabilities. Third, studies made methodological contributions that can be used to guide research with people with cognitive disabilities forward. The following sections explore these three themes in more detail.

### 5.1. Barriers to Web Accessibilty

The majority of the studies reviewed included data from interviews, surveys, focus groups, or some combination of the three aimed at gathering participant perceptions of and experiences with web accessibility. Several interesting themes arose from this type of data. Even if the website or web app met the highest WCAG standards, if it was not relevant to or immediately useful for the participants, then participants tended to dislike the website/web app or rate it poorly [30,31,32,33]. Then, beyond pure accessibility or usability, other things that are important to people with cognitive disabilities, such as opportunities to build community, share their true identities, and share expertise [34,35]. Finally, tools and platforms that serve to replicate existing social hierarchies or systems of exclusion need to be improved. Participants often cited social media sites in comments related to this theme [36,37,38].

Additional details categorised by disability type are provided below. Please note, while the overarching goal of the LIVE-IT project is to improve web accessibility for people with cognitive disabilities rather than cater to specific sub-groups of that target population, most of the studies reviewed were developed for specific target populations. Thus, accurately interpreting their results involves contextualising the studies through consideration of the characteristics of the study samples.

#### 5.1.1. Studies Involving People with Cognitive Disabilities Broadly Defined

One subset of studies involved participants described as having “intellectual disabilities,” “learning disabilities,” “cognitive disabilities,” “cognitive impairment,” and “mild cognitive impairment.” These studies tended to involve information seeking and information gathering through text-based websites. Taken together, these studies indicate that the field must devote more attention to non-text-based methods for sharing information and communicating and that PwCDs are indicating that they need more solutions anchored in real-life experiences. For example, Balasuriya, et al. [39] presented people with cognitive disabilities with web articles in “simplified” and “summarised” form and found that the use of images, familiar words, and larger font sizes influenced participants’ understanding of the information presented. Although most participants found the information easier to read, some participants noted that the experience did not satisfy their desire for knowledge because less information was provided. Karreman, et al. [40] also provided participants with two versions of a website (home page only) to pilot an easy-to-read website design, and results showed that the easy-to-read website being piloted seemed to improve participants’ efficiency in seeking and reading information. However, only a single webpage was used, which is typical of the studies reviewed and highlights the need to explore more complex situated in real-world contexts. Rocha, et al. [41] also compared multiple types of webpage navigation, text-based and image-based navigation, and results indicated that image navigation increased the speed of task-completion.

Some studies provided more in-depth views of participants’ experiences and delved further into user experience than efficiency and task completion. Such studies highlight important considerations for research design related to participant self-concept and wellbeing. For example, Moreno, et al. [42] highlighted the fact that people with cognitive disabilities often experience frustration associated with errors due to repeatedly clicking on items and perceiving delays in webpage loading as personal errors. Brunskill [43] spoke with students with learning disabilities and learned that students often relied on Google to proofread search terms, which adds nuance to the understandings of efficiency in information seeking developed in the studies presented above. Namely, the interaction with the link that takes an individual to the information needed is far from the only element in the web accessibility puzzle in online information seeking. Specific recommendations for research design related to this theme are included in Section 5.3

#### 5.1.2. Studies Involving People with Specific Cognitive Disabilities

Within the set of papers reviewed, three more specific groups of participants with cognitive disabilities appeared frequently: people with memory issues (i.e., dementia, aging, or concussion), people with autism, and people with dyslexia. Studies involving each of these different groups tended to focus on issues of web accessibility related to text. People with concussions and people with memory loss related to dementia and/or old age [44,45,46] benefit from the inclusion of audio, video, and picture modes of sharing information. Similarly, people with autism benefit from webpages with limited scrolling and direct navigation pathways [47,48]. As in the previous section, it is not necessarily a contribution of this review to point out that many studies focus on text-based websites or web applications. Rather, this finding suggests that the investigation of non-text options to support web accessibility for PwCDs is an area in need of urgent attention.

Furthermore, the majority of the studies that included a specific group of people with cognitive disabilities explored web accessibility issues for people with dyslexia [49,50,51,52,53,54,55,56,57]. Not surprisingly, these studies all explored elements of text-based webpages or web applications and highlighted a need for more graphical and/or visual presentation of online information. Overwhelmingly, the studies focused on specific elements of text, such as font size, amount of text, and text colour with an emphasis on participant accuracy and comprehension. While these are important areas of study, the focus on such areas leaves gaps in knowledge, such as the exploration of non-text solutions to web accessibility issues for PwCDs. Some studies reviewed highlight additional gaps in the literature. For example, Morris, et al. [55] show that query formulation and refining results in academic searches are barriers for students with dyslexia. Ophoff, et al. [56] noted that problems associated with the creation and management of passwords, distorted fonts on CAPTCHAs, and other login-related challenges also created persistent barriers for people with dyslexia. Such findings highlight specific areas in which non-text supports for web accessibility can be explored.

Additionally, the search returned a few studies involving people with Down syndrome, people with cerebral palsy, and people with Huntington disease [58,59,60,61]. Generally speaking, these studies focused on the usability of particular tools and/or platforms that are related to web accessibility. It is important to note that only one of these studies directly addressed web accessibility [60]. Lazar, et al. [60] provided an in-depth look at conducting design research with people with Down syndrome, which contributed to the methodological recommendations in Section 5.3. The others highlighted a particular device or a web application specifically designed for people with Down syndrome. For example, Santhanam [61] showed that using a Wii remote allowed people with cerebral palsy to more comfortably navigate webpages. Gomez, et al. [58] investigated the ways in which an Android app developed to support people with Down syndrome as they navigated workplace tasks and found that the combination of audio, visual, and haptic information supported participants in task completion. Such findings suggest avenues for continued research.

### 5.2. Tools

Of the 45 studies reviewed, 31 directly investigated websites, web applications, or web-based tools that support web accessibility for PwCDs. Many studies, 14 to be exact, either developed novel tools through the study or explored the use of novel tools created by the authors [31,38,40,41,47,50,51,58,60,62,63,64,65,66]. Additionally, 17 studies [30,32,33,34,35,36,42,45,49,53,55,56,57,62,67,68,69] investigated the potential for existing apps or web-based tools to support web accessibility for PwCDs. Existing tools examined in these studies include:

- Google apps [53];
- CAPTCHA [56];
- FindMyApps [32] (very poorly rated by users in study);
- iOS Maps [62];
- iPad Podcast [67];
- Social Support Aid [45] (very poorly rated by users in study);
- Read and Write [35];
- ICanEmail [35];
- ReACT [33] (very poorly rated by users in study);
- DigiContact [69];
- Existing Social Media Apps and Sites [30,34,36,57];
- Existing Search Engines and Databases [42,55];
- Newham Easy Read [68];
- WebHelpDyslexia [49].

Regardless of the type of website or app (built by the researcher or already existing), two common challenges arose. First, there is often too much text on the websites or web apps. Again, participant feedback across multiple studies highlighted the need for more non-text options, more real photo (as opposed to animated) options, and more audio options. Participants warned that when non-text options drastically increased the amount of scrolling required, the solution was no better than the initial problem of too much text. For example, Williams, et al. [64] developed a website that provided information primarily through audio and/or visual modes, and any benefits from interacting with a non-text-based site tended to be counteracted by the effort required to scroll through the information which expanded spatially in non-text formats. The fact that one of the few studies exploring non-text supports for web-accessibility yielded such results only emphasizes the need for continued investigation of such supports.

Second, the cognitive load associated with searching for and selecting information from the web was frequently highlighted as problematic. Participants often had to pre-search terms or phrases for use in databases to ensure correct spelling, for example. Additionally, participants reported having to toggle between multiple sites/tools/platforms to accomplish tasks without errors, relying on image searches to avoid frustration due to spelling errors, and experiencing difficulty with filtering and verifying the accuracy of search results. Such findings inform methodological recommendations made in the upcoming Section 5.3.

### 5.3. Methodological Contributions

**Figure 5:**
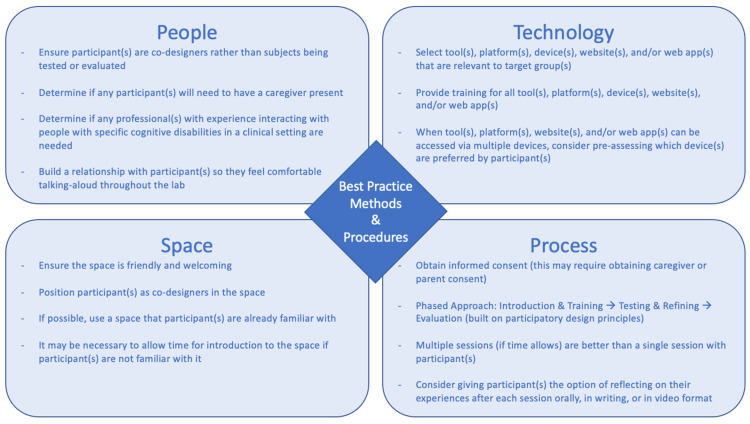
Best Practices for conducting research with people with cognitive disabilities.

The studies reviewed revealed two themes with respect to research methods. First, they provide examples of the methods used to date and the types of findings they can produce. Second, they provide a preview of what more innovative methods, such as the co-design approach the LIVE-IT labs will adopt, can bring to the field. Figure 5, below, presents a set of best practices for conducting web accessibility research with people with cognitive disabilities. Figure 5 is divided into four topics: People, Technology, Space, and Process. The recommendations related to each of these four areas are presented in Section 5.3.2.

#### 5.3.1. Methods Used to Date

Accessibility testing and usability testing that involves people with cognitive disabilities tends to involve researcher-developed websites or web applications. This happens for multiple reasons. Often the rationale is to control particular variables, to limit the number of pages, or to allow for specific modifications. There are two notable downfalls of this approach. First, this prevents the observation of challenges associated with navigating through multiple pages and/or navigating from one site to another. Second, this prevents the exploration of real-world challenges and barriers.

The majority of the studies reviewed involved text-based websites or web applications. For example, when studying information seeking, researcher-developed websites containing various levels of text-based information (simple through complex) might be used. Unfortunately, this limits the production of knowledge around how to adequately provide non-text-based methods for sharing information on the internet. Additionally, the solutions often involved presenting less material, which robs people with cognitive disabilities of developing the same sorts of understandings people who can read the complex text might develop about a particular topic.

To build websites and/or web applications, researchers often take a phased approach that involves people with cognitive disabilities at multiple stages. Initial phases often include a pilot with feedback from participants representative of the target user group. Then, revisions take place based on the pilot data. After revisions are made, the formal study takes place, which involves user testing and/or evaluations of the website or web application. In some instances, multiple design cycles take place. This mirrors the type of approach proposed for the LIVE-IT labs.

Finally, co-design and participatory design research methods tended to yield the most relevant results. What we mean by this is that the findings from studies using these methods tended to produce tools and findings that were relevant to the lives of people with cognitive disabilities. The results also tended to be anchored in real-world scenarios rather than highly controlled experimental scenarios. Such studies often involved about 10 participants who engaged in design cycles that progressed from problematizing to designing to experimenting.

#### 5.3.2. Proposed Best Practices

The concluding section of the results synthesises all findings into a set of recommended best practices. In essence, this is the state-of-the-art of research methods for studies investigating web accessibility for PwCDs. Figure 5 shows that the best practices are split into four sections: People, Technology, Space, and Process.

**People.** Based on the methods and findings presented in the 45 studies reviewed in this paper, we make four recommendations for participants in studies exploring web accessibility for PwCDs. First, ensure that participant(s) are co-designers rather than subjects being tested or evaluated. Even in instances where eye-tracking devices are employed or task completion is measured, participants should be involved in web accessibility research in ways that draw them into the research process. Second, including participants as co-designers may involve making space for caregivers in research design. Third, in a related point, including participants as co-designers may involve making space for experts in interacting with people with specific cognitive disabilities in the research design. Making space for such individuals may help support participant self-concept and wellbeing. Fourth, researchers should plan for relationship building in the research design.

**Technology.** Similarly, we developed three recommendations for making decisions related to technology in studies exploring web accessibility for PwCDs. First, select tool(s), platform(s), device(s), website(s), and/or web application(s) that are relevant to the target groups. This is an ideal space to invite participants to be co-designers. Involving PwCDs in methodological choices such as this is both empowering and a necessary step in moving towards research more grounded in real-world scenarios. Second, provide training for whatever tools are selected. This may seem to be an unnecessary inclusion, but many of the studies reviewed revealed issues with tools related to participants’ lack of familiarity with the tool. Third, consider pre-assessing what device(s) are preferred by participants, especially in instances when tools can be accessed on multiple types of devices. Again, this is an ideal space to invite participants to be co-designers.

**Space.** The recommendations for decisions related to space (i.e., lab settings or where the research is being conducted) are extensions of the recommendations related to people. First, ensure the space is friendly and welcoming. Second, position participants as co-designers in the space. These two recommendations connect with the recommendation to build relationships with participants, and they may help foster trust between a participant group that is typically marginalised and researchers. Third, consider using a space that participants are already familiar with. Since many studies cited the cognitive load associated with testing novel tools, diminishing sources of stress, such as operating in unfamiliar environments. If this is not possible, it may be necessary to allow for participants to familiarise themselves with the space.

**Process.** Finally, we make four recommendations for making decisions about research procedures. First, obtain consent, which may require obtaining caregiver, parent, or guardian consent, and repeatedly check in with participants about consent. Because PwCDs are a vulnerable population who may need accommodations to access, understand, and reply to traditional consent forms, this step may require collaboration with stakeholders to reach agreed-upon best practices. Second, consider using a phased approach to conducting research to allow time for introductions and training, testing, and refining of tools, and evaluation. A phased approach supports the inclusion of PwCDs as co-designers. Third, consider planning for multiple sessions with participants. Fourth, provide participants with the opportunity to reflect on their experiences in the research process.

## 6. Discussion

This systematic and rapid review of the literature addressing web accessibility for people with cognitive disabilities revealed three themes with implications for a variety of stakeholders. First, the studies reviewed provided insight into the web experiences of people with cognitive disabilities. Second, interventions and web-based tools used in the studies reviewed contributed to the knowledge of which tools and platforms help to support web accessibility for people with cognitive disabilities. Third, the research methods described in the studies reviewed create a foundation for best practices when conducting web accessibility research with people with cognitive disabilities. We now discuss connections to the six more specific sub-questions guiding this review. Then, we conclude the discussion by presenting implications of these results for researchers, tool and platform developers, and including people with cognitive disabilities.

### 6.1. What Are Examples of Good Practices, ‘Interventions,’ Including Examples of Platforms and Tools That Have the Potential to Support Inclusive Web Accessibility for People with Cognitive Disabilities?

The first sub-question ultimately served as the thread that connected the results of this review. Good practices for web accessibility for PwCDs are clearly defined by the W3C’s WCAG [6] and the COGA Task Force documents [8]. Much automated or expert-oriented research has been conducted concerning these best practices. Less research involving PwCDs has been conducted. Such research is siloed, meaning it is occurring in many sectors in an isolated manner. Additionally, research involving PwCDs is in need of improvement to continue building on the knowledge that has been developed in meaningful ways. Thus, another set of best practices related to research methods were developed from the studies included in this review.

### 6.2. What Is the Impact of Successful Initiatives, Interventions or Programmes on Supporting Inclusive Web Accessibility for People with Cognitive Impairment?

We include this question both in the introductory sections of this paper and here to be transparent about the nature of this review. This question aimed to gather data associated with tools or platforms (i.e., initiatives) that positively influenced web accessibility for PwCDs. Only a few of the studies included in this review convincingly tested the success of initiatives. The majority of the studies included provided more descriptive information about what tools and platforms existed. Thus, we did not fully answer this question, which points to an area for future research. The ways in which we were able to answer this question are associated with the remaining sub-questions below.

### 6.3. What Are the Similarities and Differences between Successful Initiatives, Interventions or Programmes Supporting Web Accessibility for People with Cognitive Impairment in the Different Sectors (i.e., Education, Health, Commerce, Employment, etc.)?

Throughout the results section, we describe the state-of-the-art of web accessibility for PwCDs. Similarities exist in the fact that so many of the studies focus on text-based websites or web applications. This is understandable given the fact that so much of the web is text-based. However, it highlights the need to explore non-text web-options. Differences in populations, study designs, and contexts abound. However, we had hoped to see more sectors represented in the results: education, health, commerce, employment. As shown in the introduction to the results section, the majority of the studies reviewed focus on education or web accessibility, broadly defined. It is also worth noting that many health-related studies were excluded from this review because the study population did not include PwCDs. Thus, additional research is needed in the sectors such as health, commerce, employment, and government.

### 6.4. What Are the Drivers and Barriers, or Supports and Constraints, for Web Accessibility for People with Cognitive Impairment?

Overwhelmingly, the studies reviewed presented barriers or constraints for web accessibility. Less frequently, drivers or supports for web accessibility were discussed. The text-based nature of the web and the lack of inclusion of PwCDs in design processes stand out as the major barriers to web accessibility for PwCDs. It is not a ground-breaking contribution to state that fact. However, the fact that such a statement is still a key takeaway from a literature review including work conducted as recently as 2020 highlights the importance of attending to text alternatives and methods for meaningfully including PwCDs in the research process.

### 6.5. What Are the Key Characteristics of Successful Initiatives, Interventions or Programmes on Supporting Inclusive Web Accessibility for the Population under Study?

Successful initiatives had several key characteristics. First, they were grounded in real-world or near-real-world scenarios. Second, they solicited and meaningfully incorporated feedback from PwCDs. Third, they relied on phased or cyclical approaches in the study design that allowed for multiple rounds of revision.

### 6.6. What Recommendations Can Be Made for the Future Initiatives That Wish to Support Web Accessibility for Citizens Living with Cognitive Impairment?

This sub-question, combined with several presented above, led to the development of Figure 5 and the set of best practices for conducting research with PwCDs. Our findings led to the development of best practices in four key areas: People, Technology, Space, and Process. These best practices will inform the work within the LIVE-IT Project and can also serve as a guide for others.

### 6.7. Implications for Researchers

An important implication for researchers is that they must consider innovative research designs to fully tackle the challenge of increasing web accessibility for people with cognitive disabilities. As expected, the literature highlighted barriers to web accessibility for people with cognitive disabilities. Some barriers appear to occur across the spectrum of cognitive disabilities. For example, many experience a greater cognitive load when information-seeking due to taking additional steps to vet search terms and/or determine the validity of search returns, steps not taken or completed quickly by people without cognitive disabilities. Other barriers appear to be more disability-specific, but we wonder, is that due to the design of the study or the nature of the disability? Furthermore, a large number of studies were excluded from this review due to the fact that the web accessibility testing was completely automated within the study. Studies included in the review also tended to shift to automated, or expert rather than user, accessibility testing. Such methods are relevant for gathering baseline information about barriers and informing continued research. However, we wonder what more could be learned if investments were made in research designs that fully involved people with cognitive disabilities? Questions such as these have the potential to drive research methods, and in turn research findings, forward.

A second implication for researchers is that there is an urgent need for solutions anchored in the real-world experiences of people with cognitive disabilities that involve relevant and fully functional tools. Often, accessibility testing is limited to single webpages on limited sites developed for the research project. Such sites allow for variables to be controlled, but they do not adequately represent the everyday experiences of people with cognitive disabilities. Exploratory research that allows for the capturing of more complex data is urgently needed.

### 6.8. Implications for Tool and Platform Development

The primary implication for those who design and develop web tools and platforms is related to the implications for researchers. Namely, research suggests that people with cognitive disabilities must be included in the design and testing process to move web accessibility forward in meaningful and relevant ways. Research reviewed here has shown it is both possible and productive to involve people with cognitive disabilities in all stages of the design process.

### 6.9. Implications for Including People with Cognitive Disabilities

There are also implications for people with cognitive disabilities which circle back to the implications for researchers and designers. First, it is noteworthy that web accessibility problems and solutions are primarily framed by those without disabilities. That is not to say that the researchers and designers involved in the studies reviewed are not experts in their fields and highly knowledgeable with respect to the barriers faced by people with cognitive disabilities; rather, it is to point out that if people with cognitive disabilities are not invited to take on more participatory roles within research, the findings will continue to have limited real-world and immediate implications (and usefulness) for those who stand to benefit most from them.

## 7. Conclusions

Empirical, technological, and methodological contributions to the state-of-the-art of web accessibility for people with cognitive disabilities were developed using a rapid evidence assessment approach to reviewing relevant literature. These contributions align with the calls for more robust investigations into the specific needs of people with cognitive disabilities within the campaigns for web accessibility (WCAG, 2018). Our hope is that this review provides relatively clear paths forward for such work.

## Figures and Tables

**Table 1:** Inclusion and exclusion criteria.

Component	Inclusion	Exclusion
Publication	2006–2021 Published in English in peer reviewed journals and/or full conference proceedings. Grey literature search returns presenting comprehensive reports on relevant programmes and adhere to the criteria below.	Any publication prior to 2006. Not in English, abstracts only, editorials, letters. Duplicate publications. Conference proceedings not presenting full papers.
Geographic Location	Any geographic location.	No geographic location excluded.
Study Design	All study designs.	No study design excluded.
Population	Any age 9 years and older. Experiencing cognitive impairment.	Ages younger than 9 years. Not experiencing cognitive impairment.
Intervention	All intervention types focused on the target population.	Any intervention not focused on the target population.
Comparison	All studies to be included irrespective of the presence of comparator or control group.	No exclusions based on comparison group(s).
Outcome	Data and results reported.	Studies not reporting any data or results.

**Table 2:** CASP checklist summary for qualitative, mixed-methods, and quasi-experimental studies reviewed.

CASP Checklist Question	Yes	No	Unclear
Was there a clear statement of the aim of the research?	31	0	0
Was a qualitative (or quasi-experimental or mixed methods) methodology appropriate?	31	0	0
Was the research design appropriate to address the aim of the research?	31	0	0
Was the recruitment strategy appropriate to address the aim of the research?	30	0	1
Were data generated in a way that addressed the research issues?	30	0	1
Was the relationship between researcher and participants appropriately considered?	19	0	12
Were ethical issues taken into consideration?	17	0	14
Was the data analysis sufficiently rigorous?	28	1	2
Was there a clear statement of findings?	31	0	0
	**Valuable**	**Somewhat**	**Not**
How valuable is the research?	25	6	0

**Table 3:** CASP checklist summary for experimental studies reviewed.

CASP Checklist Question	Yes	No	Unclear
Was there a clear statement of the aim of the research?	14	0	0
Was a qualitative (or quasi-experimental or mixed methods) methodology appropriate?	14	0	0
Was the research design appropriate to address the aim of the research?	14	0	0
Was the recruitment strategy appropriate to address the aim of the research?	14	0	0
Were data generated in a way that addressed the research issues?	14	0	0
Was the relationship between researcher and participants appropriately considered?	13	0	1
Were ethical issues taken into consideration?	7	0	7
Was the data analysis sufficiently rigorous?	11	2	1
Was there a clear statement of findings?	14	0	0
	**Valuable**	**Somewhat**	**Not**
How valuable is the research?	8	6	0

## Data Availability

Not applicable.

## Appendix A

**Table A1:** Full CASP Table.

Reference Number	Was There a Clear Statement of the Aim of the Research?	Was a Qualitative (or Quasi-Experimental or Mixed Methods) Methodology Appropriate?	Was the Research Design Appropriate to Address the Aim of the Research?	Was the Recruitment Strategy Appropriate to Address the Aim of the Research?	Were Data Generated in a Way That Addressed the Research Issues?	Was the Relationship between Researcher and Participants Appropriately Considered?	Were Ethical Issues Taken into Consideration?	Was the Data Analysis Sufficiently Rigorous?	Was There a Clear Statement of Findings?	How Valuable Is the Research?
[30]	Y	Y	Y	Y	Y	U	U	Y	Y	V
[31]	Y	Y	Y	Y	Y	U	U	Y	Y	V
[32]	Y	Y	Y	Y	Y	Y	U	Y	Y	S
[33]	Y	Y	Y	Y	Y	Y	Y	Y	Y	V
[34]	Y	Y	Y	Y	Y	U	U	Y	Y	V
[35]	Y	Y	Y	Y	Y	Y	U	Y	Y	V
[36]	Y	Y	Y	Y	Y	Y	Y	Y	Y	V
[37]	Y	Y	Y	Y	Y	Y	U	Y	Y	S
[38]	Y	Y	Y	Y	Y	Y	Y	Y	Y	V
[39]	Y	Y	Y	Y	Y	U	Y	Y	Y	V
[40]	Y	Y	Y	Y	Y	Y	U	N	Y	S
[41]	Y	Y	Y	Y	Y	Y	U	U	Y	S
[42]	Y	Y	Y	Y	Y	U	U	U	Y	S
[43]	Y	Y	Y	Y	Y	Y	Y	Y	Y	V
[44]	Y	Y	Y	Y	Y	Y	Y	Y	Y	V
[45]	Y	Y	Y	Y	Y	Y	Y	Y	Y	V
[46]	Y	Y	Y	Y	Y	Y	Y	Y	Y	V
[47]	Y	Y	Y	Y	Y	Y	Y	Y	Y	V
[48]	Y	Y	Y	Y	Y	Y	U	Y	Y	S
[49]	Y	Y	Y	Y	Y	U	U	Y	Y	V
[50]	Y	Y	Y	Y	Y	Y	Y	Y	Y	V
[51]	Y	Y	Y	Y	Y	U	U	Y	Y	S
[52]	Y	Y	Y	Y	Y	Y	U	Y	Y	V
[53]	Y	Y	Y	Y	Y	Y	Y	Y	Y	V
[54]	Y	Y	Y	Y	Y	Y	U	Y	Y	V
[55]	Y	Y	Y	Y	Y	Y	U	Y	Y	V
[56]	Y	Y	Y	U	U	U	U	U	Y	S
[57]	Y	Y	Y	Y	Y	U	U	Y	Y	V
[58]	Y	Y	Y	Y	Y	Y	U	Y	Y	V
[59]	Y	Y	Y	Y	Y	Y	Y	Y	Y	V
[60]	Y	Y	Y	Y	Y	Y	Y	Y	Y	V
[61]	Y	Y	Y	Y	Y	Y	Y	Y	Y	V
[62]	Y	Y	Y	Y	Y	U	U	Y	Y	S
[63]	Y	Y	Y	Y	Y	U	U	Y	Y	S
[64]	Y	Y	Y	Y	Y	Y	Y	Y	Y	V
[65]	Y	Y	Y	Y	Y	Y	Y	Y	Y	V
[66]	Y	Y	Y	Y	Y	Y	Y	N	Y	S
[67]	Y	Y	Y	Y	Y	Y	Y	Y	Y	V
[68]	Y	Y	Y	Y	Y	Y	Y	Y	Y	V
[69]	Y	Y	Y	Y	Y	U	U	Y	Y	S
[70]	Y	Y	Y	Y	Y	Y	Y	Y	Y	V
[71]	Y	Y	Y	Y	Y	Y	Y	Y	Y	V
[72]	Y	Y	Y	Y	Y	Y	Y	Y	Y	V
[73]	Y	Y	Y	Y	Y	U	Y	Y	Y	V
[74]	Y	Y	Y	Y	Y	Y	Y	N	Y	V

## Appendix B

**Table A2:** Search Strings.

Search Number	Search String
S1	(“cognitive impairment*” OR “cognitive deficit*” OR “cognitive disabilit*”) AND “web accessibility”
S2	(neurodiverse OR neurodivergent) AND “web accessibility”
S3	“complex need*” AND “web accessibility”
S4	(“cognitive impairment*” OR “cognitive deficit*” OR “cognitive disabilit*”) AND “e-services”
S5	(neurodiverse OR neurodivergent) AND “e-services”
S6	“complex need*” AND “e-services”
S7	(“cognitive impairment*” OR “cognitive deficit*” OR “cognitive disabilit*”) AND “e-commerce”
S8	(neurodiverse OR neurodivergent) AND “e-commerce”
S9	“complex need*” AND “e-commerce”
S10	(“cognitive impairment*” OR “cognitive deficit*” OR “cognitive disabilit*”) AND “e-health”
S11	(neurodiverse OR neurodivergent) AND “e-health”
S12	“complex need*” AND “e-health”
S13	(“cognitive impairment*” OR “cognitive deficit*” OR “cognitive disabilit*”) AND “e-learning”
S14	(neurodiverse OR neurodivergent) AND “e-learning”
S15	“complex need*” AND “e-learning”
S16	(“cognitive impairment*” OR “cognitive deficit*” OR “cognitive disabilit*”) AND “online learning”
S17	(neurodiverse OR neurodivergent) AND “online learning”
S18	“complex need*” AND “online learning”
S19	(“cognitive impairment*” OR “cognitive deficit*” OR “cognitive disabilit*”) AND “e-banking”
S20	(neurodiverse OR neurodivergent) AND “e-banking”
S21	“complex need*” AND “e-banking”
S22	(“cognitive impairment*” OR “cognitive deficit*” OR “cognitive disabilit*”) AND “online banking”
S23	(neurodiverse OR neurodivergent) AND “online banking”
S24	“complex need*” AND “online banking”
S25	(“cognitive impairment*” OR “cognitive deficit*” OR “cognitive disabilit*”) AND “web accessibility tool*”
S26	(neurodiverse OR neurodivergent) AND “web accessibility tool*”
S27	“complex need*” AND “web accessibility tool*”
S28	(“cognitive impairment*” OR “cognitive deficit*” OR “cognitive disabilit*”) AND “user-centered design”
S29	(neurodiverse OR neurodivergent) AND “user-centered design”
S30	“complex need*” AND “user-centered design”
S31	(“cognitive impairment*” OR “cognitive deficit*” OR “cognitive disabilit*”) AND “accessibility widgets”
S32	(neurodiverse OR neurodivergent) AND “accessibility widgets”
S33	“complex need*” AND “accessibility widgets”
S34	(“cognitive impairment*” OR “cognitive deficit*” OR “cognitive disabilit*”) AND “assistive technology”
S35	(neurodiverse OR neurodivergent) AND “assistive technology”
S36	“complex need*” AND “assistive technology”
S37	(dyslexia OR aphasia OR “non-verbal” OR “down syndrome” OR “autism” OR “dyscalculia” OR “cognitive decline” OR “attention deficit disorder” OR “learning disabilit*” OR “intellectual disabilit*” OR dysgraphia OR dementia OR “cerebral palsy” OR “brain injury” OR “motor neurone disease” OR “irlen syndrome” OR “pervasive developmental disorder”) AND “web accessibility”
S38	(dyslexia OR aphasia OR “non-verbal” OR “down syndrome” OR “autism” OR “dyscalculia” OR “cognitive decline” OR “attention deficit disorder” OR “learning disabilit*” OR “intellectual disabilit*” OR dysgraphia OR dementia OR “cerebral palsy” OR “brain injury” OR “motor neurone disease” OR “irlen syndrome” OR “pervasive developmental disorder”) AND “e-services”
S39	(dyslexia OR aphasia OR “non-verbal” OR “down syndrome” OR “autism” OR “dyscalculia” OR “cognitive decline” OR “attention deficit disorder” OR “learning disabilit*” OR “intellectual disabilit*” OR dysgraphia OR dementia OR “cerebral palsy” OR “brain injury” OR “motor neurone disease” OR “irlen syndrome” OR “pervasive developmental disorder”) AND “e-commerce”
S40	(dyslexia OR aphasia OR “non-verbal” OR “down syndrome” OR “autism” OR “dyscalculia” OR “cognitive decline” OR “attention deficit disorder” OR “learning disabilit*” OR “intellectual disabilit*” OR dysgraphia OR dementia OR “cerebral palsy” OR “brain injury” OR “motor neurone disease” OR “irlen syndrome” OR “pervasive developmental disorder”) AND “e-health”
S41	(dyslexia OR aphasia OR “non-verbal” OR “down syndrome” OR “autism” OR “dyscalculia” OR “cognitive decline” OR “attention deficit disorder” OR “learning disabilit*” OR “intellectual disabilit*” OR dysgraphia OR dementia OR “cerebral palsy” OR “brain injury” OR “motor neurone disease” OR “irlen syndrome” OR “pervasive developmental disorder”) AND “e-learning”
S42	(dyslexia OR aphasia OR “non-verbal” OR “down syndrome” OR “autism” OR “dyscalculia” OR “cognitive decline” OR “attention deficit disorder” OR “learning disabilit*” OR “intellectual disabilit*” OR dysgraphia OR dementia OR “cerebral palsy” OR “brain injury” OR “motor neurone disease” OR “irlen syndrome” OR “pervasive developmental disorder”) AND “e-banking”
S43	(dyslexia OR aphasia OR “non-verbal” OR “down syndrome” OR “autism” OR “dyscalculia” OR “cognitive decline” OR “attention deficit disorder” OR “learning disabilit*” OR “intellectual disabilit*” OR dysgraphia OR dementia OR “cerebral palsy” OR “brain injury” OR “motor neurone disease” OR “irlen syndrome” OR “pervasive developmental disorder”) AND “online learning”
S44	(dyslexia OR aphasia OR “non-verbal” OR “down syndrome” OR “autism” OR “dyscalculia” OR “cognitive decline” OR “attention deficit disorder” OR “learning disabilit*” OR “intellectual disabilit*” OR dysgraphia OR dementia OR “cerebral palsy” OR “brain injury” OR “motor neurone disease” OR “irlen syndrome” OR “pervasive developmental disorder”) AND “online banking”
S45	(dyslexia OR aphasia OR “non-verbal” OR “down syndrome” OR “autism” OR “dyscalculia” OR “cognitive decline” OR “attention deficit disorder” OR “learning disabilit*” OR “intellectual disabilit*” OR dysgraphia OR dementia OR “cerebral palsy” OR “brain injury” OR “motor neurone disease” OR “irlen syndrome” OR “pervasive developmental disorder”) AND “web accessibility tool*”
S46	(dyslexia OR aphasia OR “non-verbal” OR “down syndrome” OR “autism” OR “dyscalculia” OR “cognitive decline” OR “attention deficit disorder” OR “learning disabilit*” OR “intellectual disabilit*” OR dysgraphia OR dementia OR “cerebral palsy” OR “brain injury” OR “motor neurone disease” OR “irlen syndrome” OR “pervasive developmental disorder”) AND “user-centered design”
S47	(dyslexia OR aphasia OR “non-verbal” OR “down syndrome” OR “autism” OR “dyscalculia” OR “cognitive decline” OR “attention deficit disorder” OR “learning disabilit*” OR “intellectual disabilit*” OR dysgraphia OR dementia OR “cerebral palsy” OR “brain injury” OR “motor neurone disease” OR “irlen syndrome” OR “pervasive developmental disorder”) AND “accessibility widgets”
S48	(dyslexia OR aphasia OR “non-verbal” OR “down syndrome” OR “autism” OR “dyscalculia” OR “cognitive decline” OR “attention deficit disorder” OR “learning disabilit*” OR “intellectual disabilit*” OR dysgraphia OR dementia OR “cerebral palsy” OR “brain injury” OR “motor neurone disease” OR “irlen syndrome” OR “pervasive developmental disorder”) AND “assistive technology”
S49	(dyslexia OR aphasia OR “non-verbal” OR “down syndrome” OR “autism” OR “dyscalculia” OR “cognitive decline” OR “attention deficit disorder” OR “learning disabilit*” OR “intellectual disabilit*” OR dysgraphia OR dementia OR “cerebral palsy” OR “brain injury” OR “motor neurone disease” OR “irlen syndrome” OR “pervasive developmental disorder”)AND (“web authoring tool*” OR “web evaluation tool*”)
S50	(“cognitive impairment*” OR “cognitive deficit*” OR “cognitive disabilit*” OR neurodiverse OR neurodivergent OR “complex need*”) AND (“web authoring tool*” OR “web evaluation tool*”)
